# Known-Groups and Concurrent Validity of the Mandarin Tone Identification Test (MTIT)

**DOI:** 10.1371/journal.pone.0155595

**Published:** 2016-05-18

**Authors:** Shufeng Zhu, Lena L. N. Wong, Fei Chen, Yuan Chen, Bin Wang

**Affiliations:** 1 Department of Electrical & Electronic Engineering, Southern University of Science and Technology, Shenzhen, China; 2 Department of Electronic Engineering, Fudan University, Shanghai, China; 3 Division of Speech and Hearing Sciences, the University of Hong Kong, Hong Kong, China; 4 Neural and Cognitive Sciences Research Center, Southern University of Science and Technology, Shenzhen, China; Sun Yat-sen University, CHINA

## Abstract

**Objective:**

The Mandarin Tone Identification Test (MTIT) is a new test designed to assess the tone identification abilities of children with hearing impairment (HI). Evidence for reliability and sensitivity has been reported. The present study aimed to evaluate the known-groups and concurrent validity of the MTIT.

**Design:**

The MTIT and Mandarin Pediatric Speech Intelligibility test (MPSI) were administered in quiet and in noise conditions. The known-groups validity was evaluated by comparing the performance of the MTIT on children with two different levels of HI. The MPSI was included to evaluate the concurrent validity of the MTIT.

**Study sample:**

81 children with HI were recruited in the present study. They were Mandarin-speaking children with profound HI (mean age = 9; 0, n = 41) and with moderate to severe HI (mean age = 8; 9, n = 40).

**Results:**

Scores on the MTIT differed between the two groups with different hearing levels suggesting good known-groups validity. A strong relationship between tone and sentence perception both in quiet and in noise provided preliminary evidence for concurrent validity.

**Conclusions:**

The present study confirmed that the MTIT has good known-groups validity and provided preliminary evidence for concurrent validity. The MTIT could be used to evaluate tone identification ability in children with HI with confidence.

## Introduction

Mandarin, as a tonal language, is spoken by about 53.1% of the Chinese population [1]. The changes in fundamental frequency (F0) result in four Mandarin lexical tones: Tone 1 (high level), Tone 2 (mid-rising), Tone 3 (mid-falling-rising), and Tone 4 (high-falling) [2, 3], altering the meaning of a word. For instance, the syllable /ma/ when spoken with these four tones would respectively mean ‘mother’, ‘hemp’, ‘horse’, and ‘reproach’. The degree of contribution of lexical tones to speech understanding varies with listening conditions. For example, Chen et al. [4] found that the impact of lexical tone contour on speech understanding for normal hearing (NH) adults increased significantly in noise, although contextual cues may play a more important role than lexical tone contour when listening in quiet. Therefore, it is important to evaluate tone perception ability, especially in noise.

### Current Mandarin speech perception measures

Although there are a few standardized Mandarin speech perception measures available, few have been evaluated for psychometric properties. For example, interlist and test-retest reliability have been evaluated for the Mandarin Hearing In Noise Test (MHINT) [5]. Ding, McLoughlin and Tan [6] and McLoughlin, Xu and Song [7] have also developed two measures of tone perception in quiet and in noise. However, these tests are not appropriate for use in children with a limited vocabulary repertoire. The newly developed Mandarin Tone Identification Test (MTIT) is the only standardized tool available to evaluate lexical tone perception for children in noise [8], taking into account the vocabulary repertoire of children with hearing impairment (HI) and with stimuli designed to maintain the attention and interest of children. As adequate sensitivity, internal consistency reliability, and test-retest reliability have previously been demonstrated [8], the aims of the present study are therefore to extend the evaluation of psychometric properties to include known-groups and concurrent validity. The following is a brief review of the MTIT and evaluation of these two types of validity.

#### The Mandarin tone identification test (MTIT) [8]

The MTIT is a four-choice identification test. Although the test stimuli could be presented with three other tones of the same syllable, this method was not used because it is extremely difficult to find all four tones for each syllable but at the same time ensure that the words could be meaningful and illustrated using pictures, as well as are within the vocabulary repertoire of children with HI. Each test set of the MTIT contains one pair of monosyllable contrast (i.e., the same syllable but different tones) and one pair of unrelated distracters (i.e., both tones and syllables are different from the two contrasts). In other words, there are four words, containing the target, a tone contrast and two unrelated distracters that are chosen to decrease chance level. Plain pictures depicting these words and no words or phonetic alphabets are shown.

**Fig 1:**
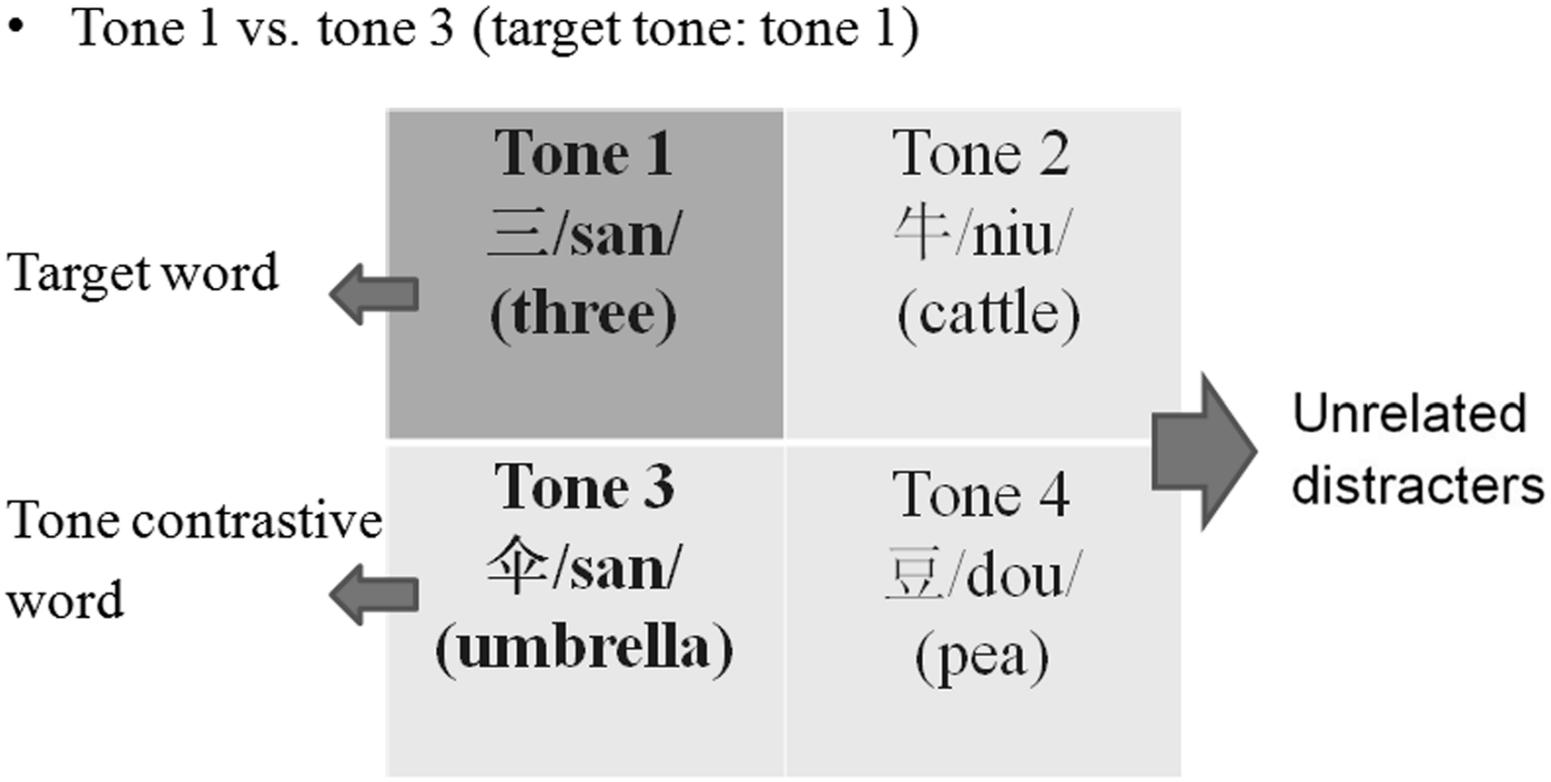
An example test set. It shows that each test set contains four pictures that are located in the four quadrants.

**Fig 2:**
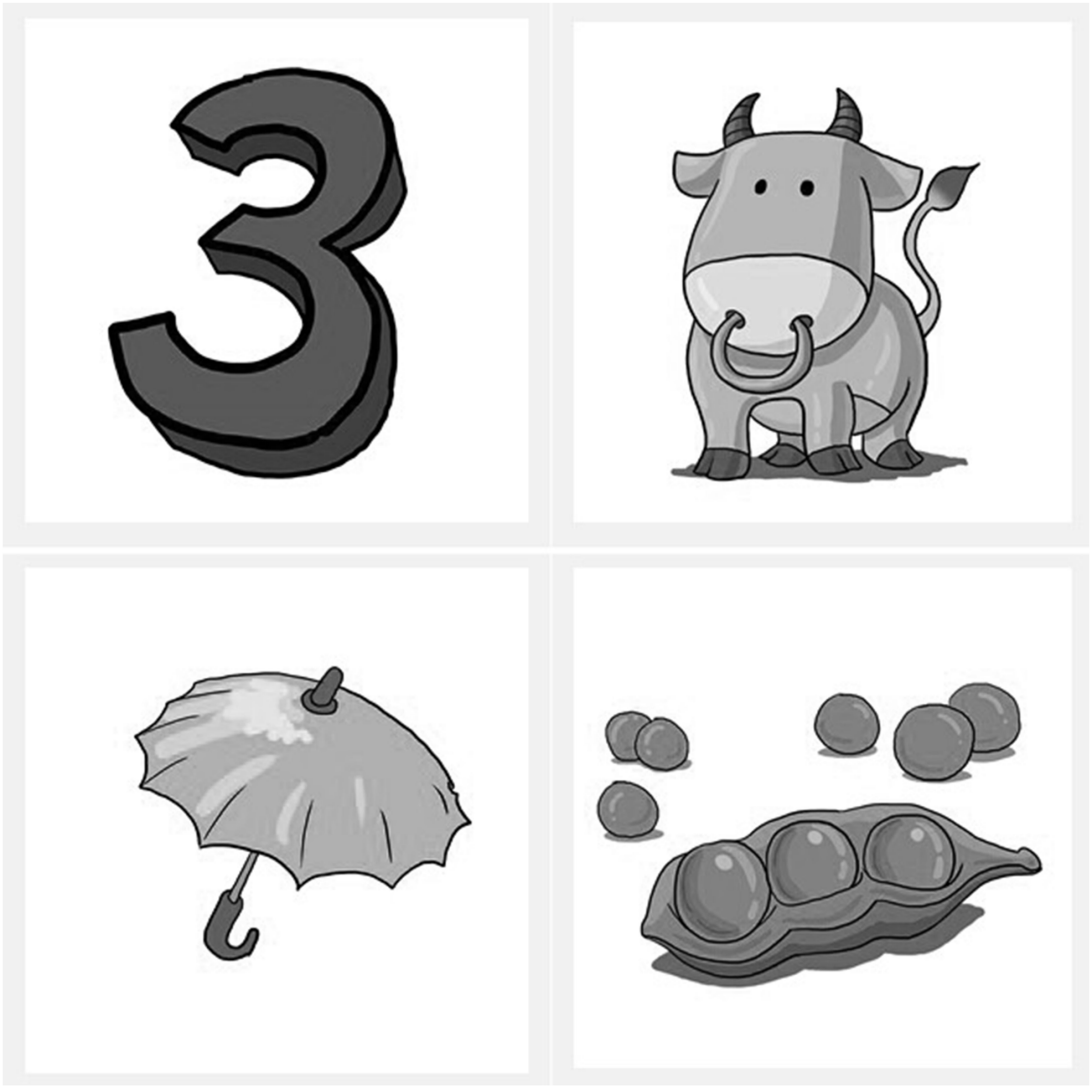
An example test set. It shows the pictorial representation of the stimuli and distractors.

A sample test set on a test plate is shown in Figs 1 and 2. Here, the target is Tone 1 (三/san/, three), while Tone 3 (伞/san/, umbrella) is a tonal contrast, and the two unrelated distracters are“牛” (/niu/, cattle, Tone 2) and “豆” (/dou /, pea, Tone 4). There are a total of 60 pairs of tone contrasts being evaluated, i.e., 6 contrastive pairs x 10 sets for each contrastive pair. Fifty-one words were used. The listener was instructed to select the target among the four choices displayed on a touch screen. The test materials in the MTIT were recorded using a male voice and are presented in conjunction with a carrier phrase. There is a one-second pause between the carrier phrase and the target word in order to avoid influences from coarticulation, and children were allowed 5 s to respond. The MTIT was conducted in quiet and in noise at -10, -5, 0 and +5 dB S/N with steady-state speech spectrum-shaped noise (SSN) as the masker. A finite impulse response filter was designed based on the average spectrum of the test words, and white noise was filtered and scaled to the same long-term average spectrum and level as the words. In each test condition, the custom program, which was developed by a professional software developer using C++ programming language, randomly selects the target and the alternatives. Percentage correct scores are obtained in each test condition.

#### Evaluation of validity

In the development of the MTIT, contrastive tone pairs were constructed to evaluate tone identification ability, ensuring face validity. In order to evaluate Mandarin tone perception, these contrastive pairs were selected by the first author from the Dictionary of Modern Chinese and major text books for Grades one and two. Only nouns and verbs that could be represented using simple pictures were selected. These monosyllables are within the daily vocabulary repertoire of children under 7 years of age.

Content validity was also verified by soliciting expert opinions [9]. That is, the words were reviewed by 16 primary school teachers and ten speech training teachers so that they are within the vocabulary repertoire of children with HI and could also be represented with simple pictures.

In the present study, other types of validity (i.e., known-groups validity and concurrent validity) were evaluated. Known-groups method was one of the approaches of evaluating construct validity [10]. A test is considered to exhibit known-groups validity if the test score could be used to discriminate between groups of participants with different features [10, 11] and in the present study, the MTIT was examined for its ability to discriminate between individuals with different degrees of hearing impairment.

Liu et al [12] found that tone perception ability varied with the degree of HI. They evaluated Mandarin tone perception in adults with three levels of hearing sensitivity: NH (pure tone average < 25 dB HL between 250 Hz and 8000Hz), mild HI (pure tone average between 26 and 45 dB HL) and moderate HI (pure tone average between 46 and 70 dB HL). They showed that Mandarin tone perception in adults with NH was better than those with mild HI and these listeners demonstrated better ability to identify tones than those with moderate HI. They attributed these differences to decreased ability to detect minor amplitude spectral changes in a tone, as the HI becomes more severe. Therefore, in the present study, known-groups validity was evaluated by examining how well the MTIT is able to distinguish tone identification scores differ across those with different degrees of unaided hearing levels.

To evaluate concurrent validity, which is a measure of criterion-related validity, MTIT results should be comparable to those obtained using a well-established test of the same nature. However, due to a lack of validated Mandarin tone perception measure, a measure that is of related nature [i.e., MPSI [13]] was used in the present study [14].

At present, there are four Mandarin tone identification tests in mainland China. That is, the Mandarin Early Speech Perception test (MESP) [15], the Mandarin tone recognition test [16], the set of Standard and Method for Evaluation of Hearing-Impaired children’s Hearing and Speech Ability [17], and the Mandarin Pediatric Lexical Tone and disyllabic-word Picture Identification Test in Noise (MAPPID-N) [18]. However, these four test materials have limitations that prevented them from serving as suitable criterion measure. First, the first three tests are to be administered in quiet and, therefore, are not expected to reflect the performance in real life situations where noise is eminently present. This is particularly important to note because speech perception in noise is more challenging, particularly for children with HI [19, 20]. Second, no carrier phrase was included in these four tests to draw children’s attention to listen and respond. The carrier phrase“请选择” (please choose) draws the children’s attention, so that they would focus on the listening task. Third and more importantly, there are few items and therefore causing uncertainties in measurement errors (i.e., these tests may not cover a range of performance and repeated measures may result in practice effects). Finally, none of these measures have psychometric properties established and therefore they could not be regarded as the standards.

As presently available measures could not be used to establish concurrent validity of the MTIT, a test that is in theory related to measures of tone perception (i.e., a sentence identification test) was used in the present study. Previous research has shown that Mandarin tone identification ability in quiet is moderately related to Mandarin sentence perception in quiet but only mildly related to sentence perception in noise in children with CI [21]. In addition, Chen et al [4] also showed that lexical tones play an important role in sentence perception in adults with NH, especially in noise. Based on these findings, tone identification scores as obtained using the MTIT should be at least moderately related to sentence perception ability in quiet and in noise for concurrent validity to be established for the MTIT.

There are two standardized measures of Mandarin sentence perception. Although the Mandarin version of the Hearing in Noise Test (MHINT) [5] was designed for normal hearing children aged 6 and above and has been evaluated for psychometric properties, the materials might be too difficult for children targeted in the present study (i.e., aged from 5 to 12 and exhibited HI) [22]. The only alternative is the Mandarin Pediatric Speech Intelligibility test (MPSI), which evaluates sentence perception in children with NH and aged below 7 years [13]. Although the psychometric properties of the MPSI has not been explicitly evaluated, the sentences were selected and judged by three schoolteachers, ensuring face and content validity. In addition, the MPSI was found to successfully discriminate children of different hearing abilities [13]. Thus, the MPSI was used to examine the concurrent validity of MTIT.

In summary, the known-groups validity and the concurrent validity of the MTIT were being evaluated. In the present study, children with different degrees of HI were recruited to assess the known-groups validity of the MTIT, and the MPSI was used as a criterion test to provide preliminary evidence for the concurrent validity of the MTIT.

## Method

In order to evaluate the known-groups validity of the MTIT, tone identification scores were obtained and compared in quiet and in noise at different signal-to-noise ratios (S/Ns) in listeners with different degrees of HI and those with NH sensitivity. Prior to the examination of concurrent validity, tone identification and sentence identification were evaluated at different S/Ns in order to select the S/N that would yield maximum sensitivity and reliability for each test. This S/N should yield the steepest performance-intensity (PI) function slope and was used to represent results obtained in noise. Results from the two tests were then correlated to examine concurrent validity.

### Materials

The speech perception test materials used in the present study include the MTIT and the MPSI. The MTIT has been introduced in the introduction. The MPSI contains two test sets and one practice set. There were three pairs of sentences in each test set and each pair had a different verb and a different object but with the same subject, such as “小鸭在坐飞机/xiao3 ya1 zai4 zuo4 fei1 ji1/ (A duck is riding in an airplane)” and “小鸭在打电话 /xiao3 ya1 zai4 da3 dian4 hua4/ (A duck is making a phone call)”. Each sentence had three key words, including one subject, one predicate and one object. Take “小鸭在坐飞机/xiao3 ya1 zai4 zuo4 fei1 ji1/ (A duck is riding in an airplane)” as an example. The three key words were “小鸭 /xiao3 ya1/ duck, (the subject)”, “坐 /zuo4/ ride, (the predicate)” and “飞机 /fei1 ji1/ airplane, (the object)”. Each sentence from the two sets was presented once in a test condition. In other words, 12 sentences were presented in a test condition. There were six test conditions, including one quiet condition and five noisy conditions (+10 dB, + 5 dB, 0 dB, -5 dB and -10 dB S/N). Percentage correct scores were obtained in each test condition. The noise was a competitive sentence different from the test sentences in that it was longer than the test sentences; and the subjects, verbs and objects were all different from test sentences. The competing sentence and the sentence stimuli were recorded using the same male voice and presented simultaneously. The words in competitive sentences are all familiar to NH children 3–4 years of age; and supposedly are familiar to children 7–10 years of age and exhibit with HI. Therefore, the materials are within the daily repertoire of children with HI in this study.

Besides MTIT and MPSI, a non-verbal intelligence test Hiskey-Nebraska Test of Learning Aptitude (H-NTLA) [23], was used to evaluate normal cognitive ability among participants. Norms are available for Chinese speaking children with NH and HI and aged from 3 to 18 [24] and the reliability and validity of the H-NTLA has been evaluated [25, 26]. There were 12 subtests, including visual memory, visual discrimination, visual organization and visual association. Participants who achieved a passing score of 84 were selected [24]. The MTIT and the MPSI were introduced above and the details could be found in Zhu et al [8] and Zheng et al [13], respectively.

### Subjects

41 children with profound HI (17 females and 24 males) and 40 children with moderate to severe HI (20 females and 20 males) were recruited from the Hearing Impairment and Rehabilitation Institute of Zhejiang Chinese Medical University in Hangzhou. Tables 1 and 2 list the details of the two groups of children. Subjects exhibited bilateral moderate to profound sloping hearing loss, without other medical comorbidities. They were all bilaterally fitted with HAs, and during testing, HAs were in users’ usual settings. That is, the HAs were adjusted to match the manufacturer prescribed settings, and modified to optimize auditory perception, according to the feedback from the participants, parents and/or teachers. They all used omnidirectional microphones without noise reduction schemes. The majority of participants (58%) had not received any rehabilitation training, which is common among children with HI in mainland China. Teachers and parents reported no learning difficulties. Language competency was not controlled in the present study because it was the intent of the current study to examine the tone and sentence perception ability in typically developing children, who may have varied language competencies. In addition, the MTIT did not require verbal responses and thus misarticulations among children, particularly those with hearing impairment, would not affect test results. Seventy-one (88%) participants were bilaterally fitted with Widex HAs. Five participants were bilaterally fitted with Resound HAs. One participant was fitted with Resound HA on one ear and Widex HA on the other ear. Two participants were bilaterally fitted with Phonak HAs and two were bilaterally fitted with Oticon HAs. They were all using digital signal processing schemes. Though different compression schemes were used in these HAs, the noise was presented 2 s before the test stimuli. The levels were maintained for the duration of the set of stimuli for the particular test condition, ensuring consistent activation of compression. However, because the majority of participants used Widex HAs, the findings of this research are probably more readily applicable to children using slow-acting compression systems.

**Table 1:** Demographics of children participated in the current study. Range of data is listed in the top row and mean and SD are listed in the bottom row for each subject group.

Group	No.	Hearing threshold (dB HL)	Chronological age (years;months)	Age of first HA fitting (years;months)	Duration of HA use (years;months)
Profound	41	81.3–110 (Mean 93.0, SD 8.6)	5;4–12;6 (Mean 9;0, SD 1;11)	0;6–11;0 (Mean 2;8, SD 2;1)	1;0–11;5 (Mean 6;4, SD 2;4)
Moderate to Severe	40	41.3–80 (Mean 63.7, SD 10.4)	5;0–12;4 (Mean 8;9, SD 1;0)	0;6–10;00 (Mean 4;7, SD 2;5)	0;1–8;11 (Mean 4;2, SD 2;2)

**Table 2:** Unaided and aided hearing thresholds of children participated in the current study.

	Unaided hearing threshold (Mean±SD dB HL)			Aided hearing threshold (Mean±SD dB HL)		
	Left ear	Right ear	Better ear	Left ear	Right ear	Better ear
Profound	96.0±9.5	97.7±9.7	**92.9±8.6**	46.1±6.8	47.2±7.5	**43.4±6.9**
moderate to severe	67.2±11.7	69.8±11.6	**63.7±10.4**	36.5±7.3	38.9±7.1	**34.1±6.5**

Note: Their unaided and aided hearing threshold averaged at 500, 1000, 2000 and 4000 Hz.

### Equipment

The test was performed in a sound booth with noise level lower than 35 dB A. Pure-tone audiometry was performed using a Madsen Midimate 622 diagnostic audiometer calibrated in accordance with ANSI S3.6 standards [27]. In each test condition, the test stimuli were presented in a randomized order using the custom program with the Realtek ALC269 sound card and via a pair of Creative Gigaworks T40 2.0 loudspeakers, placed at 0 (present target words/sentences) and 180 (present noise) degrees azimuths, respectively. The level of the two loudspeakers was at the head of the subjects and calibrated at 1 m away from the loudspeakers. For the MTIT, participants were required to choose one relative picture from four pictures on a 12-inch touch screen, and the computer recorded all responses. For the MPSI, participants were required to point to the correct picture from the six pictures displayed on a picture paper plate, and the researcher recorded the results using the custom computer software.

### Procedures

The test procedures of the MTIT and MPSI test were similar. Firstly, the participants were asked to name the materials (the targets and distracters of the MTIT and MPSI) one by one to ensure that they knew them. Practice items were then administered in quiet and in noise to ensure that the participants were familiar with the test procedures. For testing in quiet, the test stimuli level was calibrated to 65 dB A at 1 m away from the center of the head of participants. For testing in noise for both tests, the test stimuli were presented at 65 dB A, and the noise was adjusted to yield the four signal-to-noise (S/N) levels:-10, -5, 0, and +5 dB for the MTIT. For the MPSI, the noise was adjusted to yield the five S/N levels (-10, -5, 0, +5, +10 dB) for children with moderate to severe HI and four S/N levels (-5, 0, +5, +10 dB) for children with profound HI. These S/Ns were chosen based on results from a pilot test to ensure performances are at the linear portion of the PI function slope for each test. The order of test conditions was randomized across participants. For the MTIT, the participants were given 5 s to choose the picture after they heard the test stimulus. If they did not respond in 5 s, a score of zero was given for the item and the next test stimulus would be presented. The 60 test sets each took about 8–10 min to complete. Therefore, the five test conditions took about 45 min in total. For the MPSI, the response time is not limited. The participants were encouraged to respond as fast and accurate as possible. The two 6-sentence test sets each took about 4 min to complete. Therefore, the five test conditions took about 20 min in total. The MTIT and MPSI together took 100 min, including 20 min for exposing the participant to the items, 5 min for the practice, 65 min for the actual test and 10 min for the break. Research ethics was approved by the Human Research Ethics Committee for Non-Clinical Faculties of the University of Hong Kong. All participants voluntarily participated in this study with written informed consents obtained from their parents and themselves.

## Results

### Evaluation of known-groups validity

**Fig 3:**
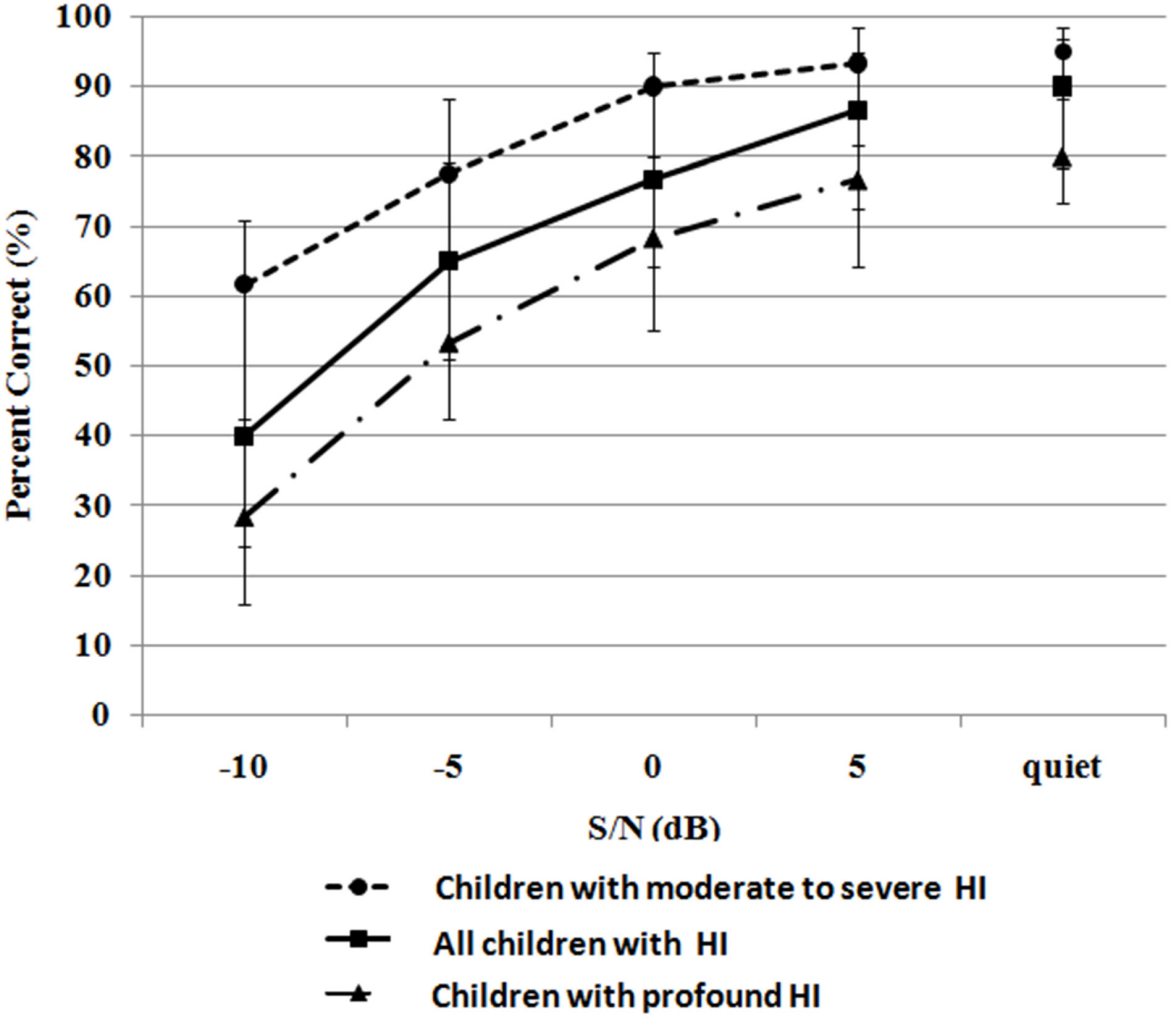
Median tone identification scores measured on the MTIT in quiet and in four noise conditions. The error bars denote interquartile range.

To verify known-groups validity of the MTIT, we examined whether tone identification scores differed significantly between listeners with different hearing levels. For children with profound HI and moderate to severe HI, MTIT median scores were plotted as a function of S/N ratios in Fig 3. MTIT scores were normally distributed after RAU transformation. Therefore, the following statistic analysis was based on RAU values. In quiet, an independent sample t test showed a significant difference in performance between the two hearing loss groups (*p*<0.001). Significant differences were also found at -10 dB S/N, -5 dB S/N, 0 dB S/N and +5 dB S/N.

For children with profound HI, one-way repeated measures ANOVA was conducted to examine the effects of five test conditions (i.e., in quiet and at four S/N levels). Mauchly’s test indicated that the assumption of sphericity was violated, χ^2^ (9) = 108.5, *p*<0.001; therefore the degree of freedom was corrected in the evaluation of the main effects of test conditions, using Greenhouse-Geisser estimates of sphericity (*ε* = .6). Results showed significant difference among the five test conditions, F (2.5, 196.3) = 333.0, *p*<0.001. Post hoc pairwise comparisons indicated significant difference across each test condition (*p*<0.001, with Bonferroni correction for multiple comparisons). For children with moderate to severe HI, the same statistical analysis was conducted to examine the effects of five test conditions (i.e., in quiet and at four S/N levels). One-way repeated measures ANOVA showed significant difference in performance among the five test conditions, F(2.9, 114.7) = 251.2, p<0.001 and post hoc pairwise comparisons showed significant difference in performance across each test condition (p<0.001, with Bonferroni correction for multiple comparisons).

Mandarin tone identification scores were also compared with results obtained in children with NH reported by Zhu et al [5]. Because NH listeners were evaluated in noise conditions (i.e., -30, -25, -20, -15 and -10 dB S/Ns), which is different from those used in the present study, a comparison of test results with those obtained in the present study could only be made at -10 dB S/N. A one-way ANOVA showed a significant main effect of hearing level on the MTIT performance in children with NH, moderate to severe HI and profound HI, *F*(3,127) = 147.07, *p*<0.001. Post hoc Games-Howell tests showed significant differences among the three groups with different hearing levels (*p*<0.05). Thus, tone identification performance of the three hearing groups differed significantly at -10 dB S/N and the performance of the two groups with HI differed significantly across the different S/Ns.

**Fig 4:**
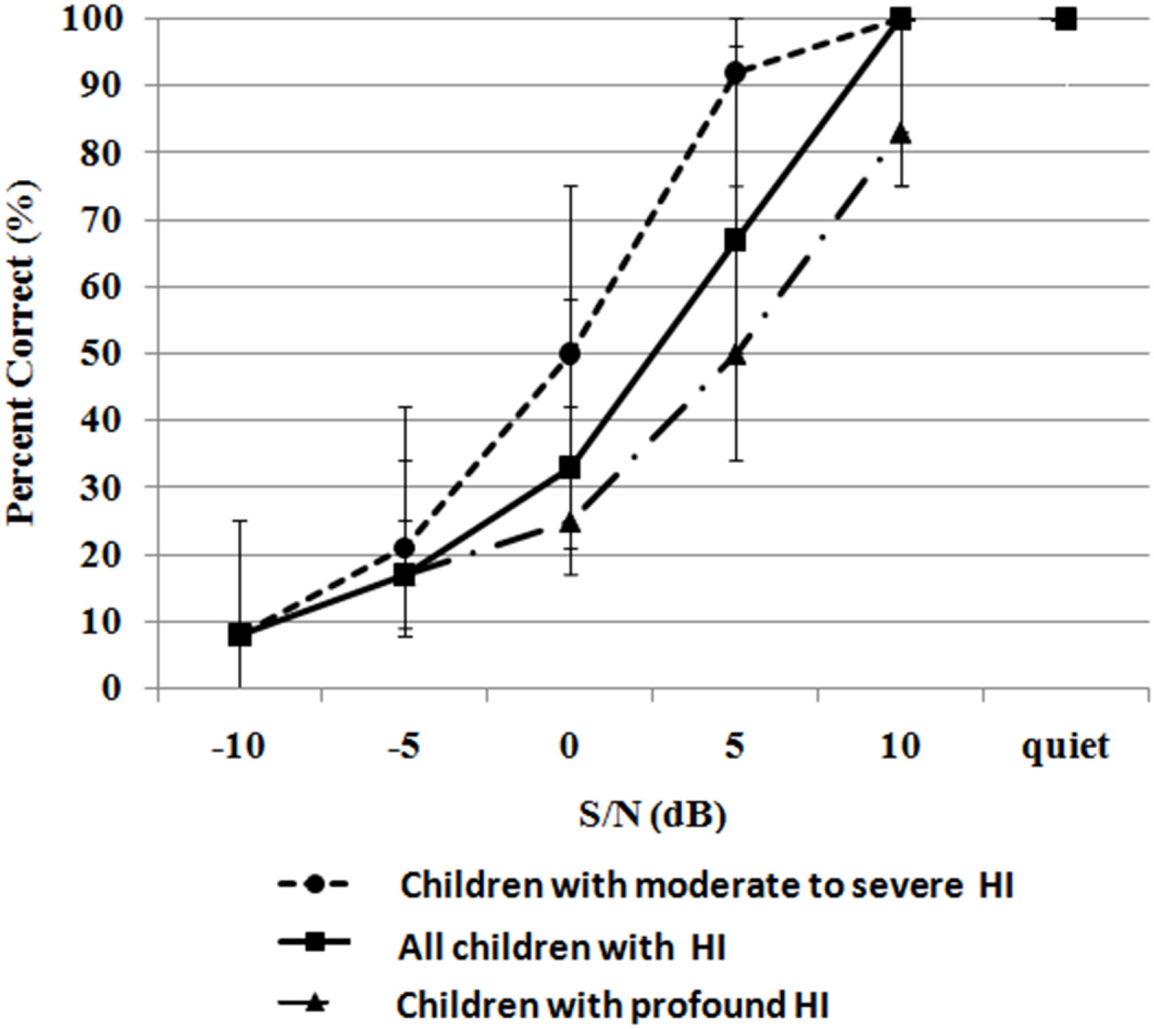
Median sentence identification scores in quiet and in five noisy conditions. The error bars denote interquartile range. Note for all children with HI: the median scores at -5, 0, 5 and 10 dB S/Ns were calculated from two groups of children with HI. Performance at -10 dB S/N was calculated from children with moderate to severe HI because the task was too difficult for children with profound HI.

To verify known-groups validity of the MPSI, we also examined whether sentence perception scores differed significantly between listeners with different hearing levels. Median scores obtained on the MPSI are plotted as a function of S/Ns in Fig 4. At -10 dB S/N and 10 dB S/N, floor and ceiling effects were noted respectively for children with moderate to severe HI. At -5 dB S/N and in quiet, floor and ceiling effects were also noted for children with profound HI. Sentence identification scores were non-normally distributed even after RAU transformation. Therefore, the Wilcoxon Mann Whitney U test was used and showed a significant difference in sentence perception ability between children with moderate to severe and with profound HI among the five test conditions (-5, 0, +5, +10 dB S/Ns and quiet) (*ps*<0.001). These results showed that the MPSI exhibits good known-groups validity.

As the data obtained on children with profound HI were not normally distributed, the Friedman test was used and yielded significant difference in performance across the five test conditions (*ps*<0.001). Post hoc comparisons using a Wilcoxon Signed Rank test showed significant difference across each test condition (*ps*<0.001). For children with moderate to severe HI, the Friedman test analysis showed significant difference across the five test conditions (*ps*<0.001). Post hoc comparisons using a Wilcoxon Signed Rank test showed significant difference across each test condition.

### Evaluation of concurrent validity

**Fig 5:**
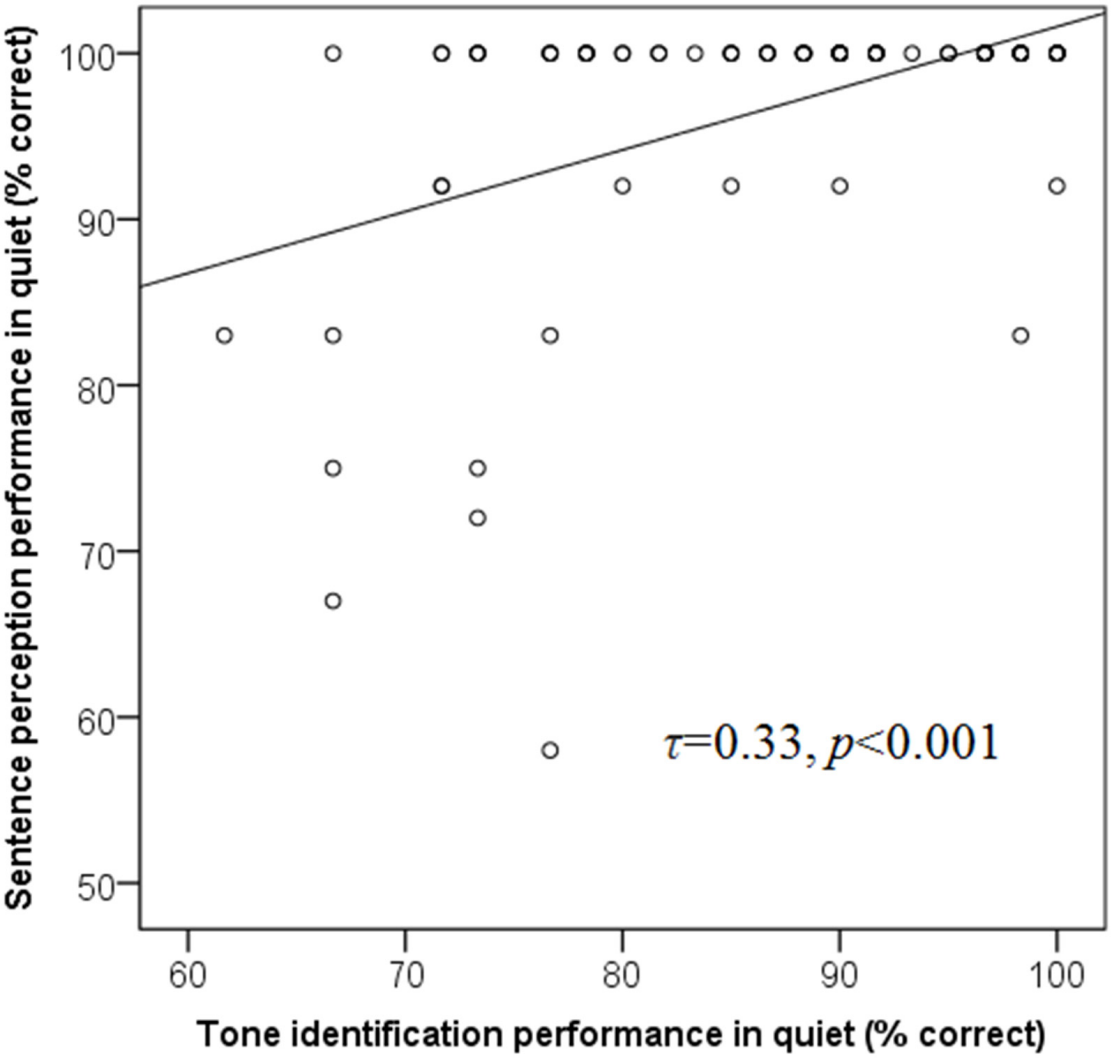
Scatter plot describing the relationship between tone identification and sentence perception in quiet, illustrated with the respective regression equation.

To evaluate concurrent validity, how well tone identification scores measured on the MTIT relate to performance obtained on a related measure (i.e., MPSI) was examined. Scores on the MPSI were not normally distributed even after RAU transformation. In addition, maximum score (100%) on the MPSI was achieved by 81% of participants in quiet at 65 dB SPL (See Fig 5). To account for scores gathered at the same rank [28, 29], Kendall’s tau was used to evaluate the relationship between tone and sentence identification and Pollock’s criterion [30] was used to determine the strength of the relationship. Therefore, Kendall’s tau (0.33) showed a strong relationship between MTIT and MPSI in quiet (See Fig 5).

**Fig 6:**
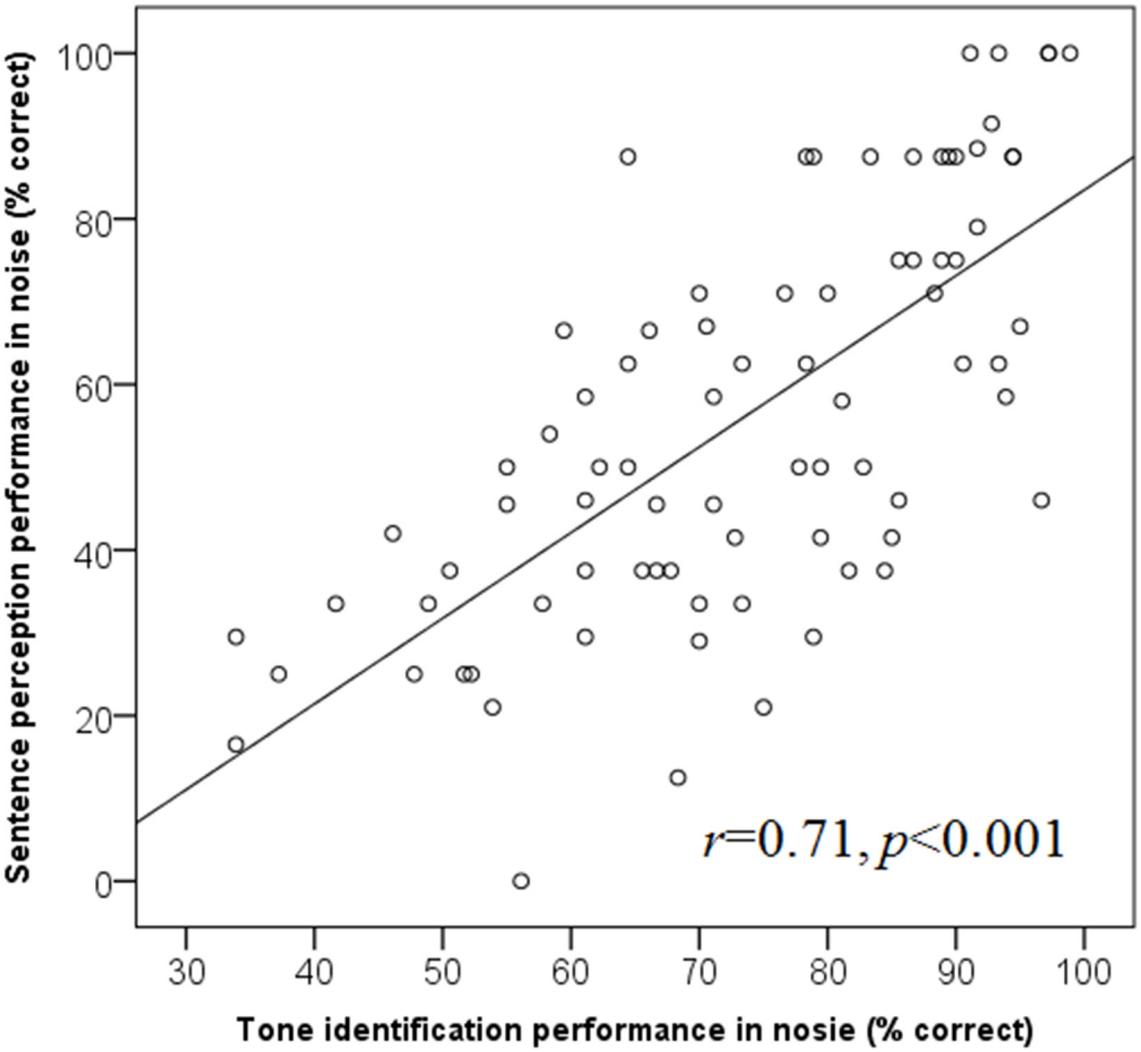
Scatter plot describing the relationship between tone identification and sentence perception in noise, illustrated with the respective regression equation.

In order to choose an appropriate condition for comparison, scores obtained in noise that are above chance level were reviewed. Those within the linear portion of the performance-intensity (PI) function were used because the performance at these S/Ns yielded steepest slopes and therefore maximum sensitivity and reliability [31]. These S/Ns were -5, 0 and +5 dB S/Ns for tone identification and 0 and +5 for sentence identification. As the data was not normally distributed, a Spearman’s rank correlation coefficient was used to evaluate the relationship between tone and sentence perception in noise and a strong significant relationship was found (*r* = 0.71, *p*<0.001) (See Fig 6).

In summary, tone identification performance measured using the MTIT and sentence perception performance measured on the MPSI was significantly related.

## Discussion

### Known-groups validity of the MTIT

The results reported above showed that tone identification scores differed across listeners with different levels of hearing and performance also differed across test conditions. Thus, the MTIT could discriminate performance among children with three different hearing levels: NH, moderate to severe HI and profound HI. Including children with mild HI in the comparison would have been ideal but was impossible because they normally would not seek intervention in mainland China. Nonetheless, these results confirmed that the MTIT has good known-groups validity, at least among those tested in the present study; future studies should include children with other levels of HI. As the participants in the current study were all native Mandarin speakers living in Zhejiang Province, further research should also examine how dialectal influences might affect known-groups validity.

### Concurrent validity of the MTIT

In the present study, the performance on the MTIT and MPSI were strongly correlated in quiet and in noise. Thus, the concurrent validity of the MTIT was verified. Further, the relationship was largely consistent with results reported previously in adults with NH [4]. That is, compared to listening in quiet, lexical tones seem to be more important for sentence identification ability in noise. Chen et al [4] used three processing methods to evaluate the effects of tone perception on Mandarin sentence intelligibility in NH adults in quiet and noise. In the first tone processing condition, the words in the sentence were presented with normal lexical tones. In the second tone processing condition, the words in the sentence were all manipulated and presented in Tone 1 contour. In the third tone processing condition, the words in the sentence were manipulated in randomly selected tone contours, from Tone 1 to Tone 4. They found that in quiet, sentence intelligibility in the first condition was 100%, 95% in the second, and 94% in the third condition. The finding revealed that in quiet, adults could still understand the sentences well even when they were presented with mismatched tones. Chen et al [4] also indicated that mistakes in the second and third conditions mainly occurred on those words with weak contextual cues, making guessing more difficult. In other words, contextual cues were important when the tones of the monosyllables were abnormal. Feng et al [32] also showed similar findings. However in noise, Chen et al [4] found that tones exhibited greater importance for identifying Mandarin sentences. Compared with the performance in quiet, the results of Mandarin sentences perception decreased significantly in noise. In addition, performance in the second and third tone processing conditions were affected more severely than the first tone processing condition. This indicated that tones play an important role in speech identification in noise, and when other cues such as context are being masked. Thus, higher correlation between tone and sentence identification in noise than in quiet provides further support for concurrent validity of the MTIT.

The current study showed a strong correlation between tone and sentence perception ability in children with HI. However, these relationships may not imply better tone perception would result in better daily communication ability. Factors such as the design and the demand of the test task may also contribute to this correlation. The sentence test used in the present study is a closed set test composing of six sentences. Compared to daily communication with varying topics, perception of the current sentence test stimuli demanded shorter attention span and lower cognitive involvement. The interpretation of the high correlation between measures should be restricted to the purpose of the present study only. While generalizing the results to real-word listening situations, we should note that other factors may contribute to the correlation between tone and sentence perception ability.

It should be noted here that different types of noise were used in the MTIT and MPSI. Speech spectrum-shaped noise (SSN), which is one of the most effective energetic maskers of speech, was used in the MTIT; competitive sentence, which is an informational masker, was used in the MPSI. Informational masker is usually equated with central masking while energetic masker is usually equated with peripheral masking [33]. Though the noises in the two tests were different, performance in SSN has been confirmed to predict well the performance in other types of noise when informational masking may be present [34]. Thus, it is not surprising to note the strong correlation between tone perception measured on the MTIT and sentence perception even when different types of noise were used.

As the present study found strong relationships between tone and sentence perception ability in children with HI both in quiet and in noise, concurrent validity of the MTIT was confirmed. The drawback of the present study was the lack of a suitable criterion tone identification test. The most appropriate speech perception test that can be used at the time of the research was the MPSI, thus the evidence for concurrent validity should be considered as preliminary.

## Conclusions

The MTIT is currently the only tone identification measure with psychometric properties verified and suitable for evaluating children five years of age and above. That is, evidence for psychometric properties, including satisfactory internal consistency reliability and good test-retest reliability, sensitivity, face validity, content validity and known-groups validity have been obtained; albeit only preliminary evidence is available for concurrent validity. The MTIT could be used for the evaluation of outcomes with hearing re/habilitation in Mandarin speaking children. The MTIT test is now available and individuals who are interested in this test could contact the first author or the Centre of Communication Disorders at the University of Hong Kong.

## Abbreviations

CIs: cochlear implants
F0: fundamental frequency
HAs: hearing aids
HI: hearing impairment
H-NTLA: Hiskey-Nebraska Test of Learning Aptitude
MTIT: Mandarin tone identification test
NH: normal-hearing
PI: performance intensity
S/N: signal to noise ratio
